# Differential experiences of embodiment between body-powered and myoelectric prosthesis users

**DOI:** 10.1038/s41598-020-72470-0

**Published:** 2020-09-22

**Authors:** Susannah M. Engdahl, Sean K. Meehan, Deanna H. Gates

**Affiliations:** 1 Department of Biomedical Engineering, University of Michigan, Ann Arbor, MI USA; 2 Department of Kinesiology, University of Waterloo, Waterloo, ON Canada; 3 School of Kinesiology, University of Michigan, Ann Arbor, MI USA

**Keywords:** Motor control, Sensorimotor processing, Human behaviour

## Abstract

Prosthesis embodiment, the perception of a prosthesis as part of one’s body, may be an important component of functional recovery for individuals with upper limb absence. This work determined whether embodiment differs between body-powered and myoelectric prosthesis users. In a sample of nine individuals with transradial limb absence, embodiment was quantified using a survey regarding prosthesis ownership and agency. The extent to which the prosthesis affected the body schema, the representation of the body’s dimensions, was assessed using limb length estimation. Because body-powered prostheses offer proprioceptive feedback that myoelectric prostheses do not, it was hypothesized that both measures would reveal stronger embodiment of body-powered prostheses. However, our results did not show differences across the two prosthesis designs. Instead, body schema was influenced by several patient-specific characteristics, including the cause of limb absence (acquired or congenital) and hours of daily prosthesis wear. These results indicate that regular prosthesis wear and embodiment are connected, regardless of the actual prosthesis design. Identifying whether embodiment is a direct consequence of regular prosthesis use would offer insight on how individuals with limb absence could modify their behavior to more fully embody their prosthesis.

## Introduction

Rehabilitation professionals who care for individuals with upper limb absence have long argued that patients who perceive a prosthesis as part of their body, rather than an auxiliary tool, might accept it more readily into their lives^1^. Unfortunately, average rejection rates for body-powered (BP) and myoelectric (MYO) prostheses are 26% and 23%, respectively^2^. These high rejection rates could suggest that patients struggle to embody their prostheses.

Embodiment of a prosthesis is a complex phenomenon that may be considered on both implicit and explicit levels^3^. An object may be implicitly embodied if some of its properties are processed in the same way as the properties of one’s own body^4^. In particular, this includes spatial properties (i.e., if the space surrounding the object is processed as body space) and motoric properties (i.e., if the object moves like a body part and is perceived to be under one’s control). Embodiment may also be experienced more explicitly, as evidenced by affective reactions towards the object, perceptions of ownership, and other subjective feelings^4^.

Both implicit and explicit embodiment of upper limb prostheses has been documented in the literature. For example, several studies have demonstrated that prosthesis users perceive their residual limb to be longer when wearing a prosthesis^5,6^ and overestimate how far they can reach with it^3^. These findings reflect changes to the body schema, or representation of one’s bodily dimensions, in response to prosthesis use. Wearing a prosthesis also expands peripersonal space boundaries outward from the residual limb to include the prosthesis^6^. Furthermore, people who wear a prosthesis frequently report an increased sense of agency over the device and demonstrate reduced sway when wearing it^7^. More explicit experiences of prosthesis embodiment have been identified through phenomenological analysis, showing that embodiment commonly involves decreased awareness of the prosthesis over time, perceptual integration of the phantom and prosthetic limbs, and viewing the prosthesis as a bodily structure rather than a tool^8^. While personal anecdotes about the perceptual experience of using a prosthesis help clarify the process through which a prosthesis may become embodied, it should be noted that these experiences occur within broader social and cultural contexts that moderate an individual’s relationship with their prosthesis^9^. Fully understanding the experience of prosthesis use requires consideration of these external factors^9^.

Regardless of what method is used to characterize embodiment, it is fundamentally dependent on the interaction between afferent and efferent signals^10^. Congruence between tactile, visual, and proprioceptive signals^11^ that are easily interpretable and concordant with a sense of agency is particularly important. The process of embodiment occurs as the brain extracts statistical correlations from multisensory inputs to create the perception that the information is arriving from a single plausible spatiotemporal source (i.e., the embodied object)^12^. Embodiment of a prosthesis also becomes more likely when a prosthesis user perceives that this sensory information comes from the interface between the residual limb, the prosthesis, and the surrounding environment^13^. Sensory feedback from a prosthesis can be delivered to the user through visual/auditory or proprioceptive/somatic pathways^14^. In BP prostheses, movement of a terminal device is activated by body movements (glenohumeral flexion or scapular abduction) via a harness and Bowden cable system. Because the state of the physiological limb is mechanically linked to the state of the prosthetic limb, the user receives information about the state of the prosthesis through the same physiological pathways that are used to activate the prosthesis^15,16^. The resulting sense of extended physiological proprioception in the prosthetic limb^17^ may minimize the conscious attention needed to control the prosthesis.

In contrast, the terminal device of a MYO prosthesis is actuated by a battery-driven motor controlled using surface electromyography signals recorded from the residual limb. The motors are typically velocity-controlled such that the output speed is directly proportional to the amplitude of the electromyography signal. Because the user is in control of the terminal device’s velocity rather than its position, they may need to constantly monitor the prosthesis visually. Indeed, studies of visuomotor behavior in upper limb prosthesis users have shown that the gaze is fixed on the hand or the area of the object to be grasped for the majority of task completion time^18,19^. However, visual feedback involves slower reaction times compared to tactile feedback^20^ and also must be consciously interpreted, placing a higher cognitive demand on the user^21^. Some sources of auditory and incidental somatic feedback (e.g., vibration from the motor, socket pressures) from the prosthesis are also available, but may not be accessible to all MYO users^22,23^.

Since prosthesis embodiment originates from the integration of multisensory inputs, but the availability of sensory input differs based on prosthesis design, it is possible that the extent of embodiment differs with prosthesis design as well. In particular, BP prostheses may be embodied more strongly than MYO prostheses since BP prostheses offer inherent proprioceptive feedback that MYO prostheses do not. Therefore, the purpose of this work was to compare the experiences of embodiment between BP and MYO prosthesis users. Embodiment was assessed using a survey about ownership and agency and a limb length estimation task to understand both implicit and explicit experiences. We hypothesized that both methods would reveal stronger embodiment of BP prostheses than MYO prostheses. To provide a reference for interpreting the performance of prosthesis users on the limb length estimation task, we also assessed the performance of individuals without upper limb absence.

## Methods

### Subjects

We recruited nine adults with unilateral transradial limb absence through the University of Michigan Orthotics and Prosthetics Center (Table 1). All participants had previous experience using an upper limb prosthesis. Three participants used BP only, three used MYO only, and three used both BP and MYO. Age- and sex-matched controls without upper limb absence were recruited from an online database (https://umhealthresearch.org/). Exclusion criteria for all participants included a history of serious neurological, visual, or musculoskeletal impairments (other than limb loss for prosthesis users). Study procedures were reviewed and approved by the Institutional Review Board of the University of Michigan Medical School. The study was carried out in accordance with the approved protocol. All participants provided written informed consent prior to participation.

**Table 1:** Participant characteristics.

ID	Age (years)	Sex	ID	Age (years)	Sex	Cause of limb absence	Affected limb	Prosthesis type	Duration of prosthesis ownership	Daily prosthesis wear	Phantom occupies same space as prosthesis?
C01	34	M	P01	32	M	Acquired	Right	BP (voluntary-open hook)	10 months	6 h	No
								MYO (iLimb)	5 months	5 h	Yes
C02	48	F	P02	52	F	Acquired	Right	BP (voluntary-open hook)	7 months	8 h	No
C03	57	F	P03	55	F	Congenital	Right	MYO (single DoF hand)	33 years	2 h	n/a
C04	63	F	P04	66	F	Congenital	Right	MYO (bebionic)	6 months	3 h	n/a
C05	23	F	P05	26	F	Congenital	Left	MYO (bebionic)	10 months	8 h	n/a
C06	29	M	P06	29	M	Acquired	Right	BP (voluntary-open hook)	2 years	10 h	Yes
C07	43	M	P07	46	M	Acquired	Right	BP (voluntary-open hook)	7 years	10 h	Yes
								MYO (iLimb)	2 years	0 h	Yes
C08	41	M	P08	54	M	Acquired	Left	BP (voluntary-close hook)	23 years	14 h	No
								MYO (single DoF hand)	23 years	6 h	No
C09*	64	M	P09	72	M	Acquired	Right	BP (voluntary-open hook)	4 months	4 h	Yes

*Data from C09 was excluded due to noncompliance with instructions.

### Surveys

Participants completed a subset of a survey about prosthesis embodiment^7^, including four questions about ownership of the prosthesis (i.e., experiencing the prosthesis as part of the body) and three questions about the sense of agency over the prosthesis (i.e., feeling control over movement of the prosthesis). All questions were scored on a five-point Likert scale, where higher scores reflect an increased sense of ownership or agency. The ownership questions and the agency questions were averaged into separate composite scores.

Satisfaction was measured using the Satisfaction with Device scale from the Orthotics and Prosthetics Users Survey (OPUS)^24^, as well as the Aesthetic Satisfaction scale, the Functional Satisfaction scale, and an 11-point Likert scale question on overall prosthesis satisfaction from the Trinity Amputation and Prosthesis Experience Scales—Revised (TAPES-R)^25^.

Participants were asked to provide several self-reported measures, including how long they had owned their prosthesis and how many hours per day they wore their prosthesis. For participants who experienced phantom limb sensations, they were also asked whether the phantom limb occupied the same space as the prosthesis when it was donned. If applicable, participants answered all questions separately for their BP and MYO prostheses.

### Limb length estimation

Participants completed a limb length estimation task^5^ in which one limb was placed inside an opaque tube (length: 91.4 cm, diameter: 13.5 cm) until it made contact with a fixed plate. Using the opposite limb, participants moved a sliding indicator along the outside of the tube to the position where they perceived the end of their hidden limb. Participants were blindfolded while they completed the task.

**Figure 1:**
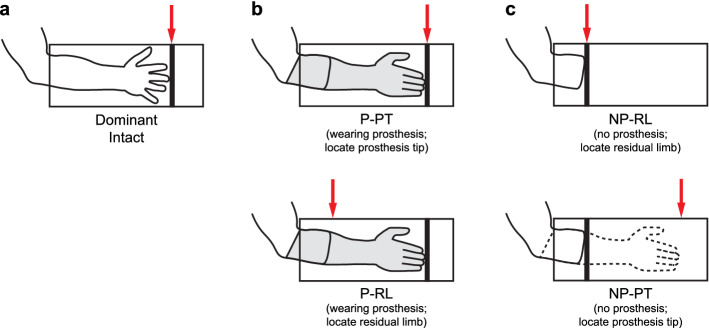
Conditions for the limb length estimation task. Participants performed the limb length estimation task by placing their arm inside an opaque tube with a sliding indicator affixed to the exterior. They performed this task using their dominant or intact limbs (**a**), their prosthetic limb (**b**), and their residual limb (**c**). For each condition, they estimated (red arrows) where they perceived the end of their hand, prosthesis (P-PT), residual limb (P-RL, NP-RL), or where the prosthesis would be if they were wearing it (NP-PT).

Control participants performed the task with their dominant (right) limb (Fig. 1a). Prosthesis users completed the task with their intact (Fig. 1a) and amputated limbs, both with and without the prosthesis. When the prosthesis was worn (Fig. 1b), participants indicated where they perceived the end of the prosthesis (P-PT) and the end of the residual limb (P-RL). When the prosthesis was not worn (Fig. 1c), participants indicated where they perceived the end of the residual limb (NP-RL) and where they imagined the end of the prosthesis would be if they were wearing it (NP-PT).

Each of these four conditions offers unique information about the participants’ perceptual adaptations to limb absence and prosthesis use. It is necessary for the user to know where the prosthesis ends when wearing it to perform functional tasks, so the P-PT condition illustrates how well their body schema has adapted to include the prosthesis. Although estimation errors of the prosthesis length indicate limitations in how the body schema has adapted, overestimation of residual limb length while wearing the prosthesis (P-RL) can actually be interpreted as evidence of embodiment. Overestimation would indicate that the residual limb is perceived to extend outwards into the space occupied by the prosthesis. This overestimation may not be retained when the prosthesis is removed, so participants were also asked to estimate residual limb length without the prosthesis (NP-RL). Importantly, removing the prosthesis also eliminates any somatosensory cues that could be used when the prosthesis is worn to help determine where it ends. As such, the NP-PT condition shows whether participants could still locate the end of their prosthesis in the absence of somatosensory cues they might normally rely on.

During the limb length estimation task, participants were asked to keep their fingers fully extended and touch the plate with their fingertips. However, some of the prosthesis users had trouble with this position either because it was difficult to fit the hand inside the tube or because the fingers passively flexed when touching the plate. In these cases, the prosthesis users were asked to make a fist instead. For prosthetic hooks or hands without finger extension, participants instead touched the plate with the most distal part of the terminal device.

For each of the four conditions, the plate was fixed at 10 randomly-chosen locations between a lower bound of 5 cm and an upper bound of the participant’s limb length (Table 2). All limb lengths were measured prior to data collection with the participant’s limb held loosely by their side. Measurements were taken from the lateral epicondyle of the humerus to the tip of the middle finger (if applicable) or the most distal point of the residual limb or terminal device. The limb length estimation error was the difference between the plate position and the indicated position, such that negative errors represented underestimation and positive errors represented overestimation.

**Table 2:** Limb lengths for prosthesis users.

ID	Intact limb length (cm)	Residual limb length (cm)	Limb length including BP prosthesis (cm)	Limb length including MYO prosthesis (cm)
P01	46.2	22.5	44.0	44.9
P02	33.6	15.9	35.4	n/a
P03	40.6	14.9	n/a	36.0
P04	43.5	11.0	n/a	33.4
P05	38.4	8.8	n/a	28.5
P06	48.0	12.0	38.0	n/a
P07	43.9	16.5	36.2	41.5
P08	46.0	14.0	42.5	42.5
P09	45.9	18.5	41.7	n/a

### Statistical analysis

To establish the validity of the limb length estimation error measure, differences in limb length estimation error between the controls’ dominant limbs and the prosthesis users’ intact limbs were assessed using unpaired t-tests. Ownership scores, agency scores, and limb length estimation error were compared between BP and MYO users with linear mixed models having the form

1 $${Y}_{ij}= \left({b}_{0}+{u}_{0j}\right)+{b}_{1}{X}_{ij}+{\epsilon }_{ij}$$

where ${X}_{ij}$ denotes the *i*th prosthesis type for the *j*th subject and ${Y}_{ij}$ is the corresponding outcome measure. In this model, ${b}_{0}$ is the intercept of the overall model, ${u}_{0j}$ is the variability of intercepts around the overall model for the *j*th subject, ${b}_{1}$ is the slope of the overall model, and ${\epsilon }_{ij}$ is the residual error. Subject was included in the model as a random effect. The variance–covariance structure for the random effects assumes all random effects are independent. All linear mixed models converged successfully. Additionally, we assessed the magnitude of the differences between BP and MYO prostheses using Hedges’ *g* as a measure of effect size:

2 $$g= \frac{{\overline{x}}_{1}-{\overline{x}}_{2}}{{s}^{*}}\left(1-\frac{3}{4\left({n}_{1}+{n}_{2}\right)-9}\right)$$

3 $${s}^{*}=\sqrt{\frac{\left({n}_{1}-1\right){{s}_{1}}^{2}+\left({n}_{2}-1\right){{s}_{2}}^{2}}{{n}_{1}+{n}_{2}-2}}$$

where s* was the pooled standard deviation weighted for sample size. Effect sizes are considered small for *g* ≥ 0.2, medium for *g* ≥ 0.5, and large for *g* ≥ 0.8^26^. For the effect size calculations, the samples were not independent because the participants using both prosthesis types were counted in each group (i.e., group 1 contained 6 instances of BP use and group 2 contained 6 instances of MYO use). Preliminary analysis of the results revealed differences in limb length estimation error between participants with congenital absence and acquired limb loss. Given the small sample size (three congenital, six acquired), we did not conduct formal statistical tests for these comparisons and report only the effect size. We also calculated Spearman rank-order correlations between limb length estimation error and self-reported measures of ownership, agency, duration of prosthesis ownership, hours of daily prosthesis wear, and satisfaction with the prosthesis.

## Results

**Figure 2:**
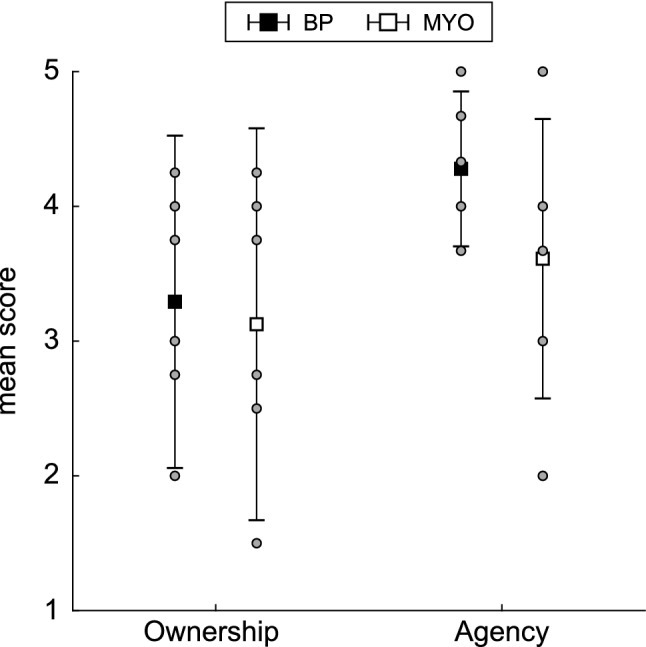
Self-reported embodiment. Mean ownership and agency scores for BP and MYO users. Error bars represent standard deviation across subjects and individual points represent individual participant scores.

Ownership scores were comparable between the two prosthesis types (*F*(1,3.49) = 0.00; *p* = 0.99; *g* = 0.11; Fig. 2). There was not a statistically significant difference in agency scores between the two prosthesis types (*F*(1, 7.73) = 2.51; *p* = 0.15), although there was a medium effect size for this comparison such that BP users had higher agency scores compared to MYO users (*g* = 0.73).

**Figure 3:**
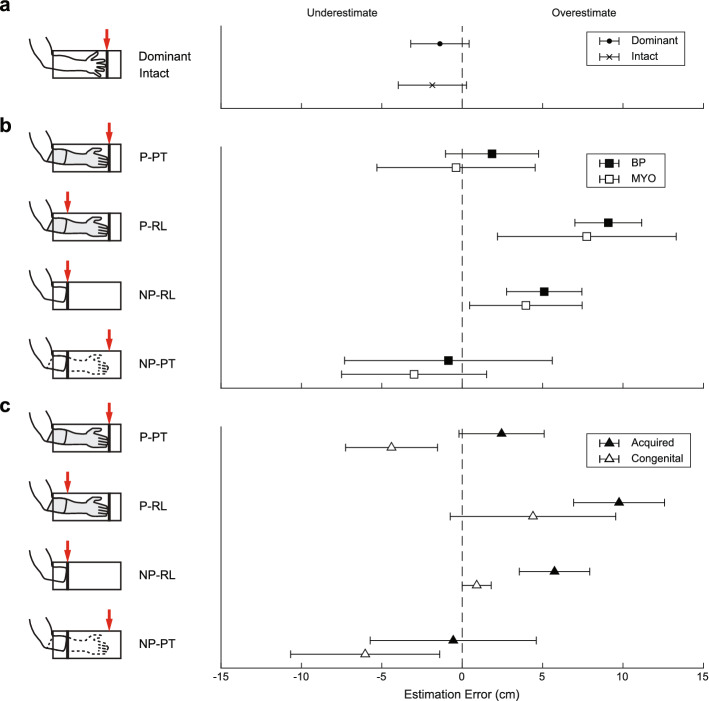
Limb length estimation error. (**a**) Average limb length estimation for the dominant (‘o’) limbs of controls, as well as the intact limbs for all prosthesis users (‘x’). (**b**) Average limb length estimation error for BP (solid squares) and MYO (open squares) prosthesis users. (**c**) Average limb length estimation error for participants with acquired limb loss (solid triangles) and congenital (open triangles) limb absence. Error bars represent standard deviation across subjects.

As expected, estimation errors were highly similar between the dominant limbs for controls and the intact limbs for prosthesis users (dominant: − 1.3 ± 2.0 cm; intact: − 1.9 ± 2.1 cm; *p* = 0.63; Fig. 3a). Estimation errors for the residual limb and prosthesis varied considerably between participants (see Supplementary Fig. S1 for individual data).

There were no significant differences in estimation error between BP and MYO prostheses (Table 3, Fig. 3b). However, there were differences in estimation error between participants with acquired limb loss and congenital limb absence for some conditions (Fig. 3c). Regardless of the prosthesis type, participants with acquired limb loss overestimated their residual limb length while wearing their prosthesis more than participants with congenital limb absence (P-RL, congenital mean: 4.4 ± 5.1 cm; acquired mean: 9.8 ± 2.8 cm; *g* = 1.31). All three participants with congenital limb absence accurately estimated their residual limb length when not wearing their prosthesis, but all participants with acquired limb loss overestimated it (NP-RL, congenital mean: 0.9 ± 0.9 cm; acquired mean: 5.7 ± 2.2 cm; *g* = 2.25). When wearing the prosthesis, the participants with congenital limb absence underestimated their prosthesis length, while the participants with acquired limb loss tended to overestimate it (P-PT, congenital mean: − 4.4 ± 2.9 cm; acquired mean: 2.4 ± 2.6 cm; *g* = 2.24). Participants with congenital limb absence also underestimated their prosthesis length while not wearing it to a greater extent than the participants with acquired limb loss (NP-PT, congenital mean: − 6.0 ± 4.6 cm; acquired mean: − 0.6 ± 5.2 cm; *g* = 0.97).

**Table 3:** Average estimation error for BP and MYO prosthesis users.

Condition	BP mean (cm)	MYO mean (cm)	F value	P value	Effect size
P-PT	1.9 ± 2.9	− 0.4 ± 4.9	F(1,1.52) = 0.38	0.62	0.51
P-RL	9.1 ± 2.1	7.7 ± 5.6	F(1,1.89) = 4.12	0.19	0.29
NP-RL	5.1 ± 2.3	4.0 ± 3.5	F(1,6.62) = 2.59	0.15	0.35
NP-PT	− 0.8 ± 6.5	− 3.0 ± 4.5	F(1,6.06) = 0.49	0.51	0.35

**Figure 4:**
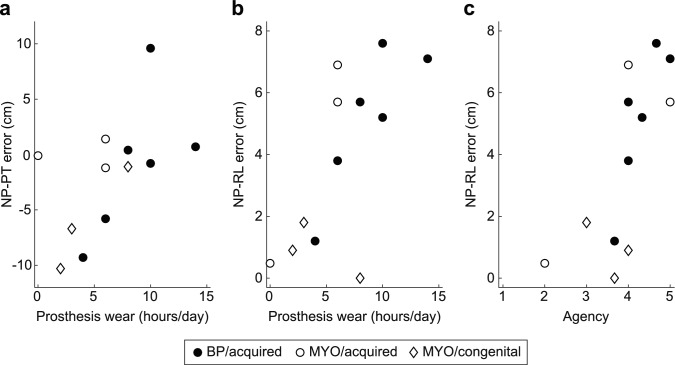
Correlations between limb length error and participant characteristics. Correlations between (**a**) NP-PT error and hours of daily prosthesis wear, (**b**) NP-RL error and hours of daily prosthesis wear, and (**c**) NP-RL and agency scores.

There was a significant positive correlation between hours of daily prosthesis wear and prosthesis length estimation error while not wearing it (ρ = 0.62; *p* = 0.03; Fig. 4a). Hours of daily prosthesis wear was also positively correlated with residual limb length estimation error while not wearing a prosthesis (ρ = 0.65; *p* = 0.02; Fig. 4b). Residual limb length estimation error while not wearing a prosthesis was positively correlated with agency as well (ρ = 0.78; *p* = 0.003; Fig. 4c). No other self-reported outcomes (i.e., ownership, duration of prosthesis ownership, and satisfaction with the prosthesis) were significantly correlated with limb length estimation error.

## Discussion

The purpose of this work was to compare experiences of embodiment between BP and MYO prosthesis users. We hypothesized that BP users would experience stronger embodiment than MYO prosthesis users due to the availability of proprioceptive feedback in BP prostheses. Our results generally failed to support this hypothesis. Rather, they suggest that the experience of prosthesis embodiment is dependent on other patient-specific characteristics beyond the type of prosthesis that is used.

There were no statistically significant differences in the sense of prosthesis ownership and agency between BP and MYO users. However, there was a trend towards a stronger sense of agency for the BP users. Ownership and agency are the result of distinct cognitive processes^27^, so it is reasonable that prosthesis users would experience these two senses differently. Ownership is thought to depend on multisensory integration^28,29^, such that spatiotemporal congruence between visual and somatic signals received from a limb allows for a sense of ownership to develop. We expected that ownership might be stronger for BP users given their purported improved access to somatic signals from the prosthesis, but our results do not support this idea. Interestingly, others have suggested that ownership is a less essential component of prosthesis embodiment in comparison to agency^7^.

In contrast, the sense of agency depends on comparison between the efferent copy and sensory feedback^30,31^. When the efferent copy and feedback match, an individual will perceive that the movement has been performed as intended and will feel a sense of agency over the movement. If the feedback does not match what is expected, the individual will fail to recognize themselves as the source of the movement^32^. Although there was not a statistically significant difference in agency between the groups, the range of scores for MYO users was much wider. In cases where MYO users have difficulty feeling agency over their prosthesis, it is possible that they are not receiving adequate sensory feedback to make a comparison with the expected outcome of their movement. Additionally, MYO users often experience uncertainty in controlling their prosthesis if the electrodes are not optimally interfaced with the residual limb during loading^33^. Any unwanted activations of the prosthesis, as well as electromechanical delays of the motors in the terminal device, may contribute to a reduced sense of agency. BP users likely experience less uncertainty when operating the prosthesis given the direct mechanical linkage between the harness and terminal device.

BP and MYO users experienced similar levels of embodiment according to the limb length estimation task. If embodiment was stronger for BP users, we might have expected to see larger overestimation error when participants estimated their residual limb length while wearing a prosthesis. Estimation error for this condition actually appears to be more dependent on the cause of limb absence (acquired or congenital) than prosthesis type. Nonetheless, the overall magnitude of our results are consistent with a previous study demonstrating that children wearing MYO prostheses overestimated their residual limb length by an average of 7.9 cm (range: 0.6–14.8 cm) when wearing their prosthesis^5^. The average overestimation error across all of our prosthesis users was similar (mean: 8.4 cm, range: 0.1–15.9 cm). McDonnell et al. did not formally report differences in overestimation error for their participants with acquired limb loss and congenital limb absence. However, the nine participants in their cohort with congenital limb absence had similar overestimation error to the three participants with acquired limb loss (congenital mean: 8.1 ± 4.3 cm, acquired mean: 7.1 ± 5.6 cm). This finding contrasts with our result that participants with acquired limb loss overestimated to a greater extent than participants with congenital limb absence. These differences could be related to participant-specific characteristics such as age or amount of prosthesis experience. The participants in McDonnell et al.’s study were children (mean: 12.3 ± 5.2 years) who may have a more malleable body schema in comparison to the adult participants from this study. Since the children’s experience with the prosthesis was unreported, it is unclear how this factor may relate to differences in study findings.

Extension of the perceived residual limb length to more closely match the prosthesis length can be interpreted as evidence of spatial embodiment^4^, as the body schema is updated to account for the dimensions of the prosthesis. Similar malleability in perceived residual limb length with prosthesis use^6^ and perceived arm length with tool use^34^ has been documented during a tactile distance perception task. In the case of tool use, it has been proposed that this malleability is a consequence of sensory feedback resulting from actions performed with the tool. When the tool contacts an external object, sensory cues are delivered to the upper limb through the tool. This creates a perceptual expansion of the space in which body-related sensory information is located^34^. Thus, the body representation expands to incorporate the tool or prosthesis so that the body is prepared to respond appropriately to stimuli that might interact with the body^6^.

It is important to note that substantial overestimation of limb length did not occur for either the prosthesis users’ intact limb or the controls’ dominant limb. For both the intact and dominant limb, there was a similar estimation of limb length. McDonnell et al. also reported that a group of 39 control subjects underestimated their limb lengths, although the dominant limbs were not analyzed separately^5^. The similarities between the intact and dominant limbs validate the estimation task and further support the idea that overestimating the residual limb length is a consequence of wearing the prosthesis, rather than a general tendency of our participants to overestimate their bodily dimensions.

An alternative explanation of the residual limb length overestimation while wearing a prosthesis could relate to the way the experiment was performed. A study of individuals with paraplegia found that they consistently overestimated their shoulder width to be closer to the width of their wheelchair^35^. Interviews following testing revealed that participants reported using the wheelchair as a reference point for estimation (i.e., visualizing the wheelchair width and subtracting to arrive at shoulder width). This strategy differs from the one employed by healthy controls, who reported using their own body as a reference point. In our study, participants were always asked to estimate their prosthesis length immediately prior to estimating their residual limb length. Because they moved the slider from the position where they perceived their prosthesis inwards towards where they perceived their residual limb, it is possible that they were conditioned to use the prosthesis as a frame of reference. However, the fact that participants with congenital limb absence underestimated their prosthesis length while wearing it contradicts this idea. This finding could indicate that they were using their residual limb as a frame of reference, thus perceiving the prosthesis to be closer to the residual limb length and shorter than it really was. As we did not ask participants what strategy they used, it is difficult to speculate further.

Despite the lack of differences between BP and MYO users, it is noteworthy that participants with congenital limb absence underestimated their prosthesis length while participants with acquired limb loss were more likely to overestimate it. This finding could reflect differences between groups in the way the body schema changes to account for whether the prosthesis is worn. Indeed, it has been repeatedly demonstrated that humans retain multiple coexisting body representations that are differentially accessed based on the current body state—with or without a prosthesis^5,6^ or tool^36–39^. For participants with acquired limb loss, the consistent overestimation of residual limb length with and without their prosthesis may reflect the ability to more readily incorporate the device into a pre-amputation body schema. In contrast, participants with congenital limb absence lack a pre-amputation schema for the residual limb. They need to generate a new body representation that incorporates the extra limb length afforded by the prosthesis, which is an experience-dependent process. For the two congenital users who had less than one year of experience using a prosthesis (P04 and P05), their ability to accurately estimate residual limb length when the device was not being worn (mean error: 1.6 ± 2.1 cm) suggests that an extended body schema for the residual limb has not been developed. In contrast, the propensity of the participant with congenital limb absence with 33 years of prosthesis experience to overestimate residual limb length (within-subject mean: 10.1 ± 2.1 cm) would suggest an extended body schema for the residual limb has been developed. This participant still retained the ability to accurately estimate residual limb length when the prosthesis was not worn, suggesting that she could transiently adopt either body schema depending on the availability of the prosthesis. Although there was no significant correlation between duration of prosthesis ownership and estimation error for this mixed sample of individuals with acquired limb loss and congenital limb absence, this relationship should be investigated in a larger sample with a greater range of prosthesis experience while accounting for the cause of limb absence.

There was also a trend towards greater underestimation of the prosthesis length when not wearing the prosthesis for participants with congenital limb absence in comparison to those with acquired limb loss. However, there was considerable variability between participants. The estimation error was quite small in seven cases (range: − 1.2 to 1.4 cm), although the error range across all participants was nearly 20 cm. It appears that this variability may be related to the amount of daily prosthesis wear, rather than just prosthesis type or cause of limb absence. In particular, the participants who used their prostheses least often tended to underestimate prosthesis length by at least 5 cm, while the more frequent users estimated more accurately. It should be noted that the magnitude of overestimation was quite large for P07, who reported using their BP prosthesis for 10 h per day and overestimated prosthesis length by 9.6 cm. Following removal of P07, the correlation coefficient maintains a similar moderate positive relationship, although it is no longer statistically significant (ρ = 0.54, *p* = 0.09). This suggests that P07 contributes to the reported relationship but is not driving it. The observed relationship between daily prosthesis wear and limb length estimation error is consistent with a previous report where prosthesis users with higher levels of prosthesis integration (which was assessed partially based on the number of hours the prosthesis was worn each day) had lower error when estimating how far they could reach with their prosthesis in comparison to the intact limb^3^. Together, these findings indicate that increased daily wear of a prosthesis improves a user’s ability to determine where the prosthesis ends, even when not actively using it.

The idea that frequent and long-term use of a prosthesis can contribute to embodiment has been supported through both phenomenological^8,9^ and behavioral^7,40^ methods. This prior work is concordant with our finding that residual limb length overestimation while wearing the prosthesis (i.e., spatial embodiment) was positively correlated with hours of daily wear. This indicator of spatial embodiment was also positively correlated with agency. A post-hoc analysis revealed that agency and hours of daily wear were also significantly correlated (ρ = 0.697, *p* = 0.01). The relationship between increased prostheses wear frequency and agency was also reported by Imaizumi et al.^7^. Taken together, these findings indicate that extensive practice with a prosthesis can contribute to both implicit and explicit embodiment.

There are several limitations to this study. First, the prosthesis length estimation errors may have been related to how prosthesis length was measured—especially for the MYO users who performed the task with a fist rather than extended fingers. The point on the prosthesis that participants were asked to locate might not have been the one that they generally used to interact with objects, so the length that they were asked to estimate may not equal the length that they actually perceive. Additionally, we did not investigate whether our participants’ experience of embodiment was influenced by the presence of phantom limb sensations, which are common among individuals with upper limb absence^41^. Similarly to intact limbs, phantom limbs have distinct spatial characteristics^42^ and can be voluntarily moved^43^ with an accompanying sense of agency that is dependent on the synchronicity of visual feedback^44^. Some individuals perceive an intersection of their prosthetic and phantom limbs such that the phantom “becomes” the prosthesis, thereby facilitating embodiment of the prosthesis^8,9^. While it is possible that phantom limb sensations could have affected the measures of embodiment used in this study, we could not explore this question in depth as only 6 participants reported experiencing phantom limb sensations (Table 1). Furthermore, embodiment was quantified using a limited set of outcome measures. Given the diversity in ways that embodiment could be measured^4^, it would be worthwhile to assess whether differences in embodiment between BP and MYO prosthesis users can be detected through other methods.

There are also several noteworthy limitations regarding the study design. In addition to the small sample size, the data set was partially paired because only three out of nine participants used both BP and MYO prostheses. Linear mixed models offer the advantage of retaining all data in estimating the model effects, whereas the unpaired samples would be discarded in a paired t-test. However, linear mixed models may be more susceptible to type I errors for small sample sizes compared to other methods, such as the optimal pooled t-test, that pool weighted estimates of variance from paired and unpaired samples^45^. A fully within-subjects comparison would have reduced confounding effects from patient characteristics such as duration of prosthesis ownership or cause of limb absence, and thus would have been the ideal design. We initially planned to only recruit people who used both devices for this reason, but it was difficult to do this in practice. Although patients are likely to benefit from having more than one type of prosthesis, prosthesis provision in the United States is often more dependent on reimbursement policies than actual patient need^46^. These reimbursement policies also vary considerably in their extent of coverage, creating unequal access to prostheses between individual patients. As such, we decided to recruit participants who have only one prosthesis in order to achieve a larger sample size.

In conclusion, this study demonstrated that the experience of prosthesis embodiment is minimally affected by the type of prosthesis that is worn. Instead, it appears that the cause of limb absence and the amount of daily prosthesis wear may play a more substantial role. Future work should explore the relationship between embodiment and a wider variety of patient-specific characteristics to better understand how prosthesis users learn to perceive, or could be trained to perceive, their prosthesis as part of their body. Ultimately, this information may contribute to an improved understanding of how individuals become functionally successful with a prosthesis.

## Supplementary information

Supplementary information.

## References

[1] Scarry E, Bender G, Druckey T (1994). The merging of bodies and artifacts in the social contract. Culture on the Brink: Ideologies of Technology.

[2] Biddiss EA, Chau TT (2007). Upper limb prosthesis use and abandonment: a survey of the last 25 years. Prosthet. Orthot. Int..

[3] Gouzien, A. *et al.* Reachability and the sense of embodiment in amputees using prostheses. *Sci. Rep.* **7** (2017). 10.1038/s41598-017-05094-6 PMC5503947 28694439

[4] de Vignemont F (2011). Embodiment, ownership and disownership. Conscious. Cogn..

[5] McDonnell PM, Scott RN, Dickison J, Theriault RA, Wood B (1989). Do artificial limbs become part of the user? New evidence. J. Rehabil. Res. Dev..

[6] Canzoneri E, Marzolla M, Amoresano A, Verni G, Serino A (2013). Amputation and prosthesis implantation shape body and peripersonal space representations. Sci. Rep..

[7] Imaizumi S, Asai T, Koyama S (2016). Embodied prosthetic arm stabilizes body posture, while unembodied one perturbs it. Conscious. Cogn..

[8] Murray CD (2004). An interpretative phenomenological analysis of the embodiment of artificial limbs. Disabil. Rehabil..

[9] Murray CD, Gallagher P, Desmond D, MacLachlan M (2008). Embodiment and prosthetics. Psychoprosthetics.

[10] Pazzaglia M, Molinari M (2016). The embodiment of assistive devices—from wheelchair to exoskeleton. Phys. Life Rev..

[11] Giummarra MJ, Gibson SJ, Georgiou-Karistianis N, Bradshaw JL (2008). Mechanisms underlying embodiment, disembodiment and loss of embodiment. Neurosci. Biobehav. Rev..

[12] Armel KC, Ramachandran VS (2003). Projecting sensations to external objects: evidence from skin conductance response. Proc. R. Soc. Lond. B. Biol. Sci..

[13] Mills FB (2013). A phenomenological approach to psychoprosthetics. Disabil. Rehabil..

[14] Childress DS (1973). Powered limb prostheses: their clinical significance. IEEE Trans. Biomed. Eng..

[15] Weir RF, McCombs KP (2003). Design of artificial arms and hands for prosthetic applications. Standard Handbook of Biomedical Engineering and Design.

[16] Antfolk C (2013). Sensory feedback in upper limb prosthetics. Expert Rev. Med. Devices.

[17] Simpson, D. The choice of control system for the multimovement prosthesis: extended physiological proprioception (epp). In *The Control of Upper-Extremity Prostheses and orthoses* 146–150 (1974).

[18] Bouwsema H, Kyberd PJ, Hill W, van der Sluis CK, Bongers RM (2012). Determining skill level in myoelectric prosthesis use with multiple outcome measures. J. Rehabil. Res. Dev..

[19] Sobuh MM (2014). Visuomotor behaviours when using a myoelectric prosthesis. J. Neuroeng. Rehabil..

[20] Nelson RJ, McCandlish CA, Douglas VD (1990). Reaction times for hand movements made in response to visual versus vibratory cues. Somatosens. Mot. Res..

[21] Gonzalez J, Soma H, Sekine M, Yu W (2012). Psycho-physiological assessment of a prosthetic hand sensory feedback system based on an auditory display: a preliminary study. J. Neuroeng. Rehabil..

[22] Sörbye R (1980). Myoelectric prosthetic fitting in young children. Clin. Orthop. Relat. Res..

[23] Wijk U, Carlsson I (2015). Forearm amputees' views of prosthesis use and sensory feedback. J. Hand Ther..

[24] Orthotics prosthetics user survey. *Rehabilitation Measures Database* https://www.sralab.org/rehabilitation-measures/orthotics-prosthetics-users-survey (2015).

[25] Gallagher P, Franchignoni F, Giordano A, MacLachlan M (2010). Trinity amputation and prosthesis experience scales: a psychometric assessment using classical test theory and rasch analysis. Am. J. Phys. Med. Rehabil..

[26] Cohen J (1988). Statistical Power Analysis for the Behavioral Sciences.

[27] Kalckert, A. & Ehrsson, H. Moving a rubber hand that feels like your own: A dissociation of ownership and agency. *Front. Hum. Neurosci.* **6** (2012). 10.3389/fnhum.2012.00040 PMC3303087 22435056

[28] Makin TR, Holmes NP, Ehrsson HH (2008). On the other hand: dummy hands and peripersonal space. Behav. Brain Res..

[29] Ehrsson HH, Stein BE (2012). The concept of body ownership and its relation to multisensory integration. The New Handbook of Multisensory Processes.

[30] Blakemore S-J, Frith C (2003). Self-awareness and action. Curr. Opin. Neurobiol..

[31] Haggard P (2017). Sense of agency in the human brain. Nat. Rev. Neurosci..

[32] Farrer C, Bouchereau M, Jeannerod M, Franck N (2008). Effect of distorted visual feedback on the sense of agency. Behav. Neurol..

[33] Chadwell A, Kenney L, Thies S, Galpin A, Head J (2016). The reality of myoelectric prostheses: understanding what makes these devices difficult for some users to control. Front. Neurorobot..

[34] Canzoneri E (2013). Tool-use reshapes the boundaries of body and peripersonal space representations. Exp. Brain Res..

[35] Arnhoff FN, Mehl MC (1963). Body image deterioration in paraplegia. J. Nerv. Ment. Dis..

[36] Cardinali L (2009). Tool-use induces morphological updating of the body schema. Curr. Biol..

[37] Serino A, Bassolino M, Farne A, Ladavas E (2007). Extended multisensory space in blind cane users. Psychol. Sci..

[38] Farnè A, Làdavas E (2000). Dynamic size-change of hand peripersonal space following tool use. NeuroReport.

[39] Maravita A, Iriki A (2004). Tools for the body (schema). Trends Cogn. Sci..

[40] Fraser C (1984). Does an artificial limb become part of the user?. Br. J. Occup. Ther..

[41] Kooijman CM, Dijkstra PU, Geertzen JH, Elzinga A, van der Schans CP (2000). Phantom pain and phantom sensations in upper limb amputees: an epidemiological study. Pain.

[42] Longo MR, Long C, Haggard P (2012). Mapping the invisible hand: a body model of a phantom limb. Psychol. Sci..

[43] Raffin E, Mattout J, Reilly KT, Giraux P (2012). Disentangling motor execution from motor imagery with the phantom limb. Brain.

[44] Imaizumi, S., Asai, T., Kanayama, N., Kawamura, M. & Koyama, S. Agency over a phantom limb and electromyographic activity on the stump depend on visuomotor synchrony: a case study. *Front. Hum. Neurosci.* **8** (2014). 10.3389/fnhum.2014.00545 PMC4114199 25120449

[45] Guo B, Yuan Y (2017). A comparative review of methods for comparing means using partially paired data. Stat. Methods. Med. Res..

[46] Jette AM, Spicer CM, Flaubert JL, National Academies of Sciences, E., and Medicine (2017). Upper-extremity prostheses. The Promise of Assistive Technology to Enhance Activity and Work Participation.

