# Supplementary material for: Differential experiences of embodiment between body-powered and myoelectric prosthesis users
Source: Sci Rep. 2020 Sep 22;10:15471. doi: 10.1038/s41598-020-72470-0 (PMC7508812; doi:10.1038/s41598-020-72470-0)
Supplement: Supplementary file 1 — Supplementary information. [file 41598_2020_72470_MOESM1_ESM.pdf]

# Differential experiences of embodiment between body-powered and myoelectric prosthesis users

Susannah M. Engdahl, Sean K. Meehan, Deanna H. Gates

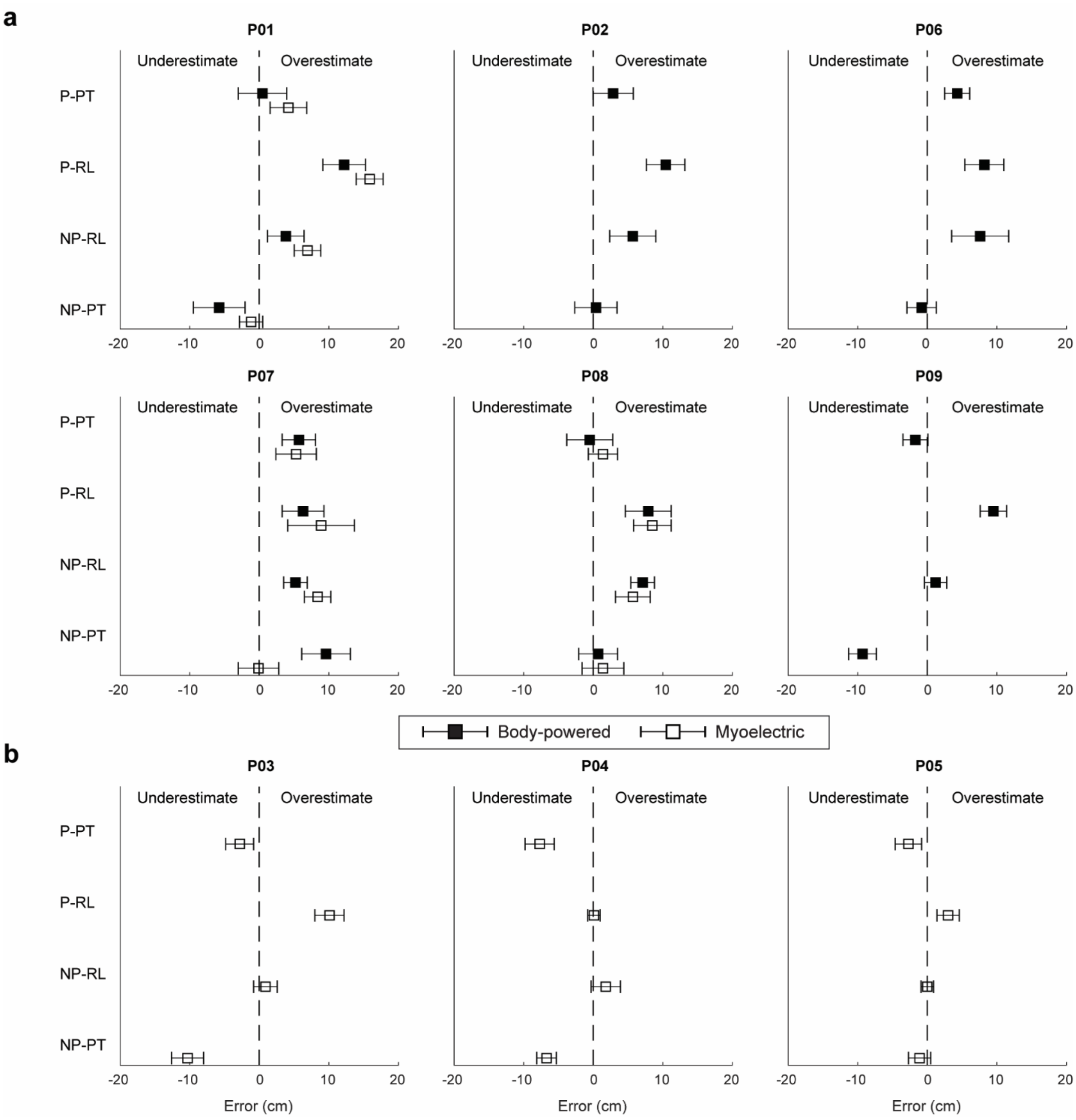


**Supplementary Figure 1.** Average limb length estimation error for individual participants with (**a**) acquired or (**b**) congenital limb absence using body-powered (solid squares) or myoelectric (open squares) prostheses. Error bars represent within-subject standard deviation.
